# Prevalence of collagen VII-specific autoantibodies in patients with autoimmune and inflammatory diseases

**DOI:** 10.1186/1471-2172-13-16

**Published:** 2012-04-04

**Authors:** Emilia Licarete, Susanne Ganz, Martin J Recknagel, Giovanni Di Zenzo, Takashi Hashimoto, Michael Hertl, Giovanna Zambruno, Gheorghe Hundorfean, Jonas Mudter, Markus F Neurath, Leena Bruckner-Tuderman, Cassian Sitaru

**Affiliations:** 1 Department of Dermatology, University of Freiburg, Hauptstr. 7, Freiburg 79104, Germany; 2 Department of Experimental Biology and Molecular Biology Center, Institute for Interdisciplinary Research on Bio-Nano-Sciences, Babes-Bolyai University Cluj-Napoca, Cluj-Napoca, Romania; 3 Faculty of Biology, Genetics and Experimental Bioinformatics Group, University of Freiburg, Freiburg, Germany; 4 Molecular and Cell Biology Laboratory, IDI-IRCCS, Rome, Italy; 5 Department of Dermatology, Kurume University, 67 Asahimachi, Kurume, Fukuoka 830-0011, Japan; 6 Department of Dermatology and Allergology, University of Marburg, Baldingerdstraße, Marburg 33043, Germany; 7 University of Erlangen-Nuremberg, Medical Clinic I, Erlangen, Ulmenweg 18, Erlangen 91054, Germany; 8 Centre for Biological Signalling Studies (bioss), University of Freiburg, Freiburg, Germany

## Abstract

**Background:**

Autoimmunity to collagen VII is typically associated with the skin blistering disease epidermolysis bullosa acquisita (EBA), but also occurs occasionally in patients with systemic lupus erythematosus or inflammatory bowel disease. The aim of our present study was to develop an accurate immunoassay for assessing the presence of autoantibodies against collagen VII in large cohorts of patients and healthy donors.

**Methods:**

Based on *in silico *antigenic analysis and previous wetlab epitope mapping data, we designed a chimeric collagen VII construct containing all collagen VII epitopes with higher antigenicity. ELISA was performed with sera from patients with EBA (n = 50), Crohn's disease (CD, n = 50), ulcerative colitis (UC, n = 50), bullous pemphigoid (BP, n = 76), and pemphigus vulgaris (PV, n = 42) and healthy donors (n = 245).

**Results:**

By ELISA, the receiver operating characteristics analysis yielded an area under the curve of 0.98 (95% CI: 0.9638-1.005), allowing to set the cut-off at 0.32 OD at a calculated specificity of 98% and a sensitivity of 94%. Running the optimized test showed that serum IgG autoantibodies from 47 EBA (94%; 95% CI: 87.41%-100%), 2 CD (4%; 95% CI: 0%-9.43%), 8 UC (16%; 95% CI: 5.8%-26%), 2 BP (2.63%; 95% CI: 0%-6.23%), and 4 PV (9.52%; 95% CI: 0%-18.4%) patients as well as from 4 (1.63%; 95% CI: 0%-3.21%) healthy donors reacted with the chimeric protein. Further analysis revealed that in 34%, 37%, 16% and 100% of sera autoantibodies of IgG1, IgG2, IgG3, and IgG4 isotype, respectively, recognized the recombinant autoantigen.

**Conclusions:**

Using a chimeric protein, we developed a new sensitive and specific ELISA to detect collagen specific antibodies. Our results show a low prevalence of collagen VII-specific autoantibodies in inflammatory bowel disease, pemphigus and bullous pemphigoid. Furthermore, we show that the autoimmune response against collagen VII is dominated by IgG4 autoantibodies. The new immunoassay should prove a useful tool for clinical and translational research and should improve the routine diagnosis and disease monitoring in diseases associated with collagen VII-specific autoimmunity.

## Background

An immune response against collagen VII is typically associated with epidermolysis bullosa acquisita (EBA) and bullous systemic lupus erythematosus, but may occur in other conditions, including inflammatory bowel disease (IBD) and dystrophic epidermolysis bullosa [1,2]. EBA is an acquired subepidermal blistering disease of the skin and mucous membranes associated with an autoimmune response to collagen VII [3,4]. EBA is characterized by bound and circulating IgG autoantibodies which label the dermal side of split skin by direct and indirect immunofluorescence (IF) microscopy, respectively [5-7]. Accumulating clinical and experimental evidence demonstrates that collagen VII-specific IgG autoantibodies are pathogenic. Transient skin blistering was reported in a newborn from a mother with EBA showing the transplacental transfer of pathogenic autoantibodies [8]. IgG autoantibodies from EBA patients induced dermal-epidermal separation in frozen sections of normal human skin when co-incubated with granulocytes from healthy donors [9]. Further *in vivo *work showed that the passive transfer of collagen VII-specific antibodies into mice induced subepidermal blisters [10]. Immunization with autologous collagen VII induces a T cell-dependent autoimmune response and subepidermal blisters in mice [11-13].

Collagen VII, the main structural component of the anchoring fibrils, is a 290 kDa protein composed of three identical α chains, each consisting of a central collagenase sensitive triple helical portion flanked by a 145 kDa N-terminal (NC1) and a 34 kDa C-terminal (NC2) non-collagenous domains [14,15]. Two molecules of collagen VII associate through a small overlap of the C-terminal NC2 domain resulting in the dimer form present in anchoring fibrils. In the extracellular space, large part of the NC2 domain is proteolytically removed by bone morphogenetic protein 1 (BMP-1), an enzyme with procollagen C-proteinase activity. In spite of this proteolytic cleavage a small peptide of NC2 domain consisting of 41 aminoacids still reside in the dermis, below the lamina densa [16-18]. Epitope mapping studies revealed that the major epitopes recognized by EBA autoantibodies reside within the NC1 domain of native collagen VII [19,20]. In addition to very few cases showing reactivity to the triple helical domain of collagen VII, further important epitopes of EBA autoantibodies have been more recently mapped to the NC2 domain [21,22]. The laboratory diagnosis of EBA relies on several laboratory tests, including detection of tissue-bound autoantibodies by direct IF microscopy and demonstration of serum autoantibody binding to the dermal side of the 1 M salt-split skin by indirect IF microscopy. The definitive diagnosis of EBA requires characterization of the molecular specificity of autoantibodies [1]. Autoantibodies against collagen VII are commonly detected by immunoblotting and/or ELISA using recombinant proteins [1]. While collagen VII-specific autoantibodies are present in virtually all EBA patients, their exact prevalence in patients with IBD or other autoimmune bullous diseases is still controversial [23,24] or unknown, respectively.

For detection of collagen VII-specific IgG autoantibodies by ELISA, immunoassays using recombinant forms of the NC1 domain or the full-length molecule have been developed [22,25-27]. Based on B cell epitope *in silico *prediction and wetlab mapping studies, a recombinant form of collagen VII containing both the NC1 and NC2 domains as well as the hinge region would allow a most sensitive detection of anti-collagen VII antibodies in patients. In addition, expression of a lower molecular mass non-collagenous protein should allow for better protein yields compared with the expression of the full-length collagenous molecule. Therefore, in the present study, we have developed an immunoassay for the detection of collagen VII-specific autoantibodies using a chimeric recombinant fusion protein composed of both non collagenous domains and the hinge region of collagen VII. This protein, expressed in stably transfected HEK-293 cells to ensure optimal posttranslational modifications, contains all putative epitopes of collagen VII in equimolar amounts and was utilized to develop a sensitive ELISA for the detection in the same assay of EBA autoantibodies. Using this immunoassay the prevalence of collagen VII-specific autoantibodies and their IgG subclass was characterized in large cohorts of patients with IBD, pemphigus vulgaris (PV) and bullous pemphigoid (BP) as well as healthy donors.

## Methods

### Human sera

Serum samples were obtained from patients with EBA (n = 50), Crohn's disease (CD; n = 50), ulcerative colitis (UC; n = 50), BP (n = 76), and PV (n = 42) before initiation of treatment and healthy donors (n = 245). EBA and BP patients were characterized by: (a) subepidermal skin blisters, (b) linear IgA or IgG deposits along the dermal-epidermal junction detected by direct IF microscopy, and (c) circulating IgG autoantibodies binding to the epidermal (BP) or dermal (EBA) side of the salt-split skin as revealed by IF microscopy. PV was diagnosed in patients presenting (a) intraepidermal skin blisters and mucosal or mucocutaneous involvement, (b) intercellular IgG deposits within the epidermis detected by direct immunofluorescence (DIF) microscopy, (c) Serum IgG autoantibodies binding to the epithelium of monkey esophagus with an intercellular pattern by IF microscopy, and (d) IgG autoantibodies against desmoglein 3 by ELISA. Sera from patients with a moderate or severe UC or CD were included. The patients were selected within the IBD section of the Medical Clinic I Erlangen, University of Erlangen-Nuremberg, having a confirmed diagnosis for an IBD entity based on the consensus evidence-based clinical, endoscopical and histopathological criteria [28]. The study was approved by the Ethics Committee of the Medical Faculty of the University of Freiburg, Germany (Institutional Board Projects no 318/07, 425/08 and 278/11). We obtained informed consent from patients whose material was used in the study, in adherence to the Helsinki Principles.

### Cell culture

Transfected Flp-In HEK 293 T cells (Flp-In™-293, Invitrogen) were cultured in DMEM medium, with phenol red (Lonza) supplemented with 10% FCS, L-glutamine, penicillin, streptomycin (all from Biochrome). When cells reached 70% confluence, complete growth medium was replaced with serum free medium supplemented with 100 μg/ml of vitamin C. After 48 hours, cell culture medium was collected, cleared by centrifugation at 3200 × g for 7 min at 4°C and 5 mM EDTA and 1 mM PMSF were added. The supernatant was stored at -20°C until used.

### Generation of recombinant noncollagenous domains of human collagen VII

cDNA sequences corresponding to the non-collagenous domains of human collagen VII (NC1, NC2) were obtained by polymerase chain reaction (PCR) amplification on 8xHis tagged full length collagen VII sequence (kind gift from A Fritsch), previously cloned into prokaryotic vector pcDNA3.1 Zeo(-), Invitrogen [29]. Primers for PCR were synthesized by Eurofins MWG (Ebersberg, Germany;Table 1). Restriction sites for EcoRI and HindIII were introduced by primers (Table 1). Briefly, pcDNA3.1hcol7 vector containing the full length sequence of human collagen VII was digested with EcoRI and AgeI restriction enzymes. The digested vector containing the sequence spanning aminoacids 1-443 was ligated with the PCR fragment overlapping the restriction site for AgeI within collagen VII sequence resulting in the recombinant vector pcDNA3.1hCol7NC1 containing the entire sequence of NC1 domain of collagen VII spanning the aminoacids 1-1278. The PCR product corresponding to the NC2 fragment was digested with EcoRI and HindIII restriction enzymes and ligated into pcDNA3.1hCol7NC1 vector digested with the same enzyme resulting in the recombinant vector pcDNA3.1hCol7NC1-NC2 with the sequence spanning the aminoacids 1-1278 and 2776-2944. Subsequently, the recombinant fragment (NC1-NC2) was subcloned into the pcDNA5FRT vector with CMV promoter using NheI and HindIII restriction enzymes resulting pcDNA5FRThcol7NC1-NC2 recombinant vector. To obtain the recombinant protein containing NC1, hinge region and NC2 domains of type VII collagen the nucleotide sequence coding the hinge region and NC2 domain, flanked by the restriction sites for EcoRI and Hind III, was synthesized by GenScript in pUC57 vector. Further, the sequence was cut out with EcoRI and HindIII restriction enzymes and ligated into the pcDNA5FRT NC1-NC2 vector digested with the same enzymes resulting pcDNA5FRThcol7NC1-H-NC2 recombinant vector containing the sequence spanning the aminoacids 1-1278, 1940-1979 and 2776-2944. Correct DNA sequences of all vectors were confirmed by direct secquencing. Flp-in Hek293 T host cells were transfected with 5 μg of pcDNA5FRThCol7NC1-NC2/pcDNA5FRThCol7NC1-H-NC2 and 2.5 μg of pOG44 vectors in lipofectamine2000 (Invitrogen). Transfected cells expressing the desired proteins were selected under 200 μg/ml hygromicine (Roth). Proteins were precipitated from the culture medium with 50% ammonium sulphate for 4 h at 4°C and collected by centrifugation at 27000 × g for 45 minutes at 4°C. The proteins were resuspended in cold PBS, dyalised overnight against PBS and purified by metallochelate affinity chromatography using nickel nitrilotriacetic acid coupled with agarose (Ni-NTA, Qiagen, Germany). Purified proteins were separated by SDS PAGE on 8% gels under reducing conditions and transferred on nitrocellulose membrane. Membrane strips were incubated with 1000-fold diluted monoclonal antibody specific for human collagen VII (clone LH 7.2; Chemicon International, Germany) and reactivity was detected with secondary, HRP-conjugated goat anti-mouse IgG antibodies (Abcam).

**Table 1:** Primer sequences for PCR amplification of col7a1 cDNA fragments

Fragment	Size (bp)	Primer sequences (5'-3')
NC1	2573	FP: GATCCTGGGCCCCACATCCATCCTC

		RP: GATCGAATTCGCCCGGGAGGCCAGGGTCG

NC2	524	FP: GATCGAATTCGGCGAGAAGGGAGAAGCTGC

		RP: GATCAAGCTTTCAGTCCTGGGCAGTACCTGT

FP forward primer, RP reverse primer

### Enzyme-linked immunosorbent assays

ELISA was developed and performed using previously established protocols with modification [30,31]. Briefly, 96-well microtiter plates (Greiner Bio-One, Germany) were coated with 500 ng/well of recombinant His-hCVII-NC1-NC2 and an equimolar amount of His-hCVII-NC1-H-NC2 in 0.1 M bicarbonate buffer (pH 9.6), overnight at 4°C. Next day the plates were washed with 0.05% Tween20-PBS (w/v) and blocked 1 h with 1% BSA-PBS (w/v) followed by incubation with 1:100 diluted sera in 1% BSA-0.05%Tween20-PBS (w/v) for 1 h. Bound antibodies were detected by 1 hour incubation with a mixture of 2000-fold diluted biotin conjugated mouse antibodies recognizing the four human IgG subclasses (Invitrogen) and subsequently with horseradish-peroxidase conjugated Streptavidin (Dianova) diluted 1:250. After washing, color reaction was developed by addition of orthophenylene diamine substrate (Dako). Reaction was stopped after 10 minutes with 0.5 M sulphuric acid solution. All steps were carried out at room temperature. The optical density (OD) was read at 492 nm using an automated spectrophotometer (Sirius HT-TRF, MWG). Each serum was tested in triplicate. The cut-off for positivity was validated and optimized by receiver-operating characteristics (ROC) analysis as described below. The accuracy of the assay was expressed as sensitivity = true positive/(true positive + false negative) and specificity = true negative/(true negative + false positive).

### SDS-PAGE and immunoblot analysis

Immunoblotting with recombinant proteins was performed as described with minor modification [31]. Briefly, preparations of recombinant His-hCVII-NC1-NC2 and His-hCVII-NC1-H-NC2 proteins were separated by SDS-PAGE on 8% preparative gels, under reducing conditions, followed by transfer onto nitrocellulose (Whatman/Protran BA85). Membrane strips were incubated with 100-fold diluted EBA and normal human sera. Reactivity was visualized with secondary, HRP-conjugated goat anti-human IgG antibodies (Abcam) and diaminobenzidine (Merck).

### Indirect immunofluorescence

Serum IgG autoantibodies were detected by IF following published protocols [31]. Briefly, frozen sections of salt-split healthy human skin were incubated in a first step with serially diluted sera. IgG antibodies bound at the dermal side were visualized with 100-fold diluted, Alexa Fluor488-labelled polyclonal goat anti-human IgG antibody (Invitrogen).

### In silico and statistical analysis

Linear and conformational epitopes on collagen VII were analyzed *in silico *using software available at 3 different web servers. BepiPred predicts the location of linear B-cell epitopes using a combination of a hidden Markov model and a propensity scale method protocol http://www.cbs.dtu.dk/services/BepiPred/[32]. CBTOPE predicts conformational B-cell epitope of an antigen from its amino acid sequence (http://www.imtech.res.in/raghava/cbtope/) [33]. The Predictor component of Epitope Toolkit (EpiT; http://ailab.cs.iastate.edu/bcpreds/) was used to predict flexible length linear B-cell epitopes by the FBCPred algorythm [34]. A ROC curve allows for exploring the relationship between the sensitivity and specificity of the ELISA for a variety of different cut-off points, thus allowing the determination of an optimal cut-off point for positivity. Therefore, to determine the cut-off value for the ELISA using recombinant forms of type VII collagen, we performed a ROC analysis by plotting on the X-axis the 1 - specificity (the false positive rate) and on the Y-axis the sensitivity (the true positive rate). Statistical analyses were performed using the GraphPad Prism statistical package (v5; GraphPad Software, San Diego, CA). Statistical significance was calculated using the non-parametric Mann-Whitney-*U *test and correlations were analyzed by the Spearman's rank correlation test; p < 0.05 was considered significant.

## Results

### *In silico *prediction of B cell epitope

**Figure 1:**
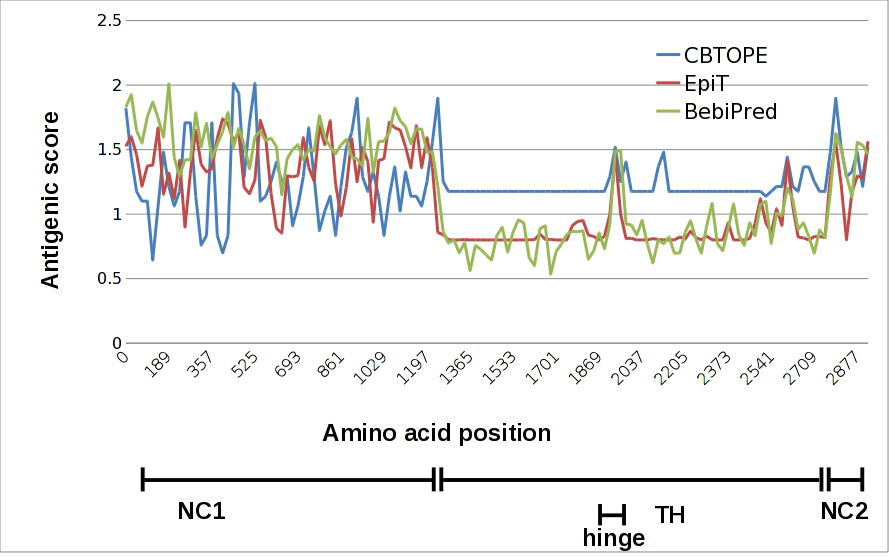
***In silico *analysis of B cell epitopes on human collagen VII**. Linear and conformational antigenic determinants of collagen VII were analyzed *in silico *using BepiPred (http://www.cbs.dtu.dk/services/BepiPred/), CBTOPE (http://www.imtech.res.in/raghava/cbtope) and the Predictor component of Epitope Toolkit (EpiT; http://ailab.cs.iastate.edu/bcpreds/). The antigenic scores are plotted for the entire sequence of human collagen VII. The different regions of the autoantigen, including its non-collagenous (NC) 1 and 2 domains as well as the triple helical (TH) and hinge regions are shown in the lower part of the figure.

Wet lab epitope mapping studies using different recombinant fragments of collagen VII showed that the epitopes targeted by autoantibodies are localized within the NC1, NC2 and, possibly, the hinge region of the antigen. To further our understanding about the distribution of B cell epitopes on collagen VII, we applied *in silico *analysis of both linear and conformational epitopes using different algorithms. The results revealed that NC1 and NC2 domains as well as the hinge region within the triple helix of collagen VII contain more antigenic sites compared with its collagenous domain (Figure 1, Additional file 1: Table S1).

### Generation of the recombinant forms of the autoantigen

**Figure 2:**
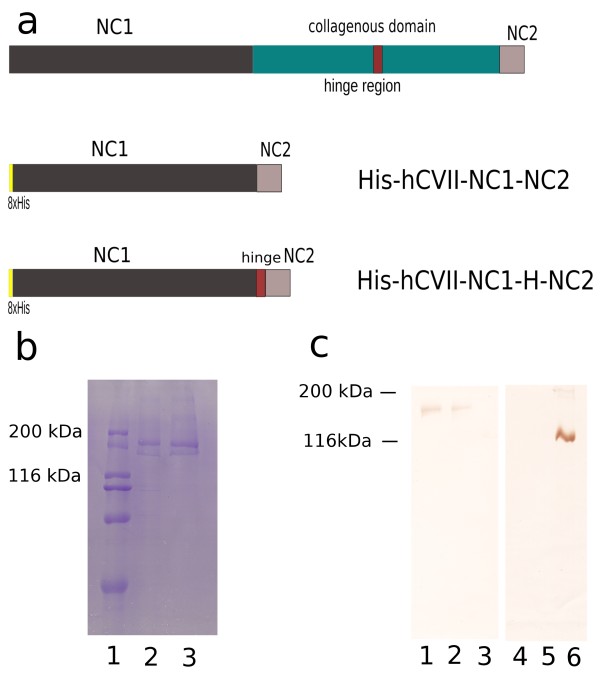
**Recombinant forms of collagen VII used in this study**. **(a) **Schematic representation of human collagen VII consisting of a central collagenous domain flanked by a large, 145 kDa N-terminal non-collagenous domain and a smaller, 30 kDa non-collagen domain at its C-terminus. The collagenous domain is interrupted by a 39 amino acid non-collagenous hinge region. The recombinant forms of collagen VII generated in this study are two N-terminally 8xhistidine tagged chimeric proteins termed His-hCVII-NC1-NC2 and His-hCVII-NC1-H-NC2 corresponding to the fused NC1 and NC2 domains (aa 1-1278, 2776-2944) and to the fused NC1, hinge and NC2 regions (1-1278, 1940-1979, 2776-2944) of the antigen, respectively. **(b) **Sodium dodecylsulfate-polyacrylamide gel electrophoresis of the purified recombinant His-hCVII-NC1-NC2 and His-hCVII-NC1-H-NC2 proteins shows their migration at around 153 (lane 2) and 158 kDa (lane 3), respectively. Weight markers of 200, 116, 97, 66 and 45 kDa are shown in lane 1. **(c) **Immunoblot analysis of the two recombinant proteins His-hCVII-NC1-NC2 (lanes 1 and 4) and His-hCVII-NC1-H-NC2 (lanes 2 and 5) as well as a recombinant form of collagen XVII [47] (lanes 3 and 6) using a monoclonal antibody specific to the NC1 domain of collagen VII (clone LH7.2; lanes 1-3) and a monoclonal antibody specific to soluble ectodomain of collagen XVII [47] (lanes 4-6).

The recombinant proteins were expressed in mammalian cells and purified by metallochelate affinity chromatography. When separated by SDS-PAGE, the recombinant collagen VII forms containing the NC1 fused with the NC2 domain (His-hCVII-NC1-NC2) as well as the hinge region (His-hCVII-NC1-H-NC2), migrated consistently with their calculated molecular masses of 153 kDa (Figure 2b, lane 2) and 158 kDa (Figure 2b, lane 3), respectively. A monoclonal antibody specific for the NC1 domain of collagen VII recognized both recombinant forms by immunoblot analysis (Figure 2c, lanes 1 and 2).

### Immunoreactivity of recombinant collagen VII with EBA autoantibodies

**Figure 3:**
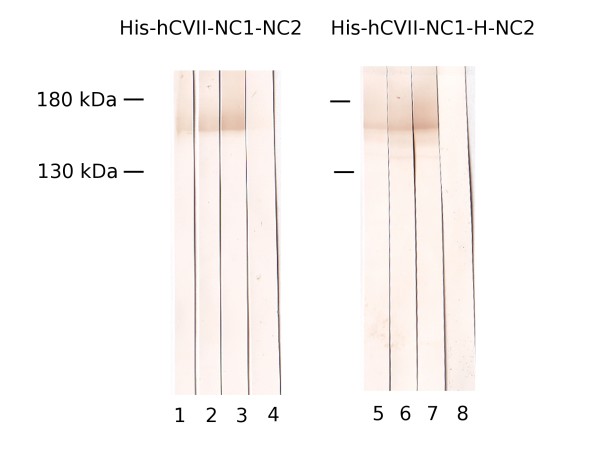
**Immunoreactivity of epidermolysis bullosa acquisita (EBA) autoantibodies with the recombinant forms of collagen VII**. Purified, recombinant His-hCVII-NC1-NC2 (lanes 1-4) and His-hCVII-NC1-H-NC2 (lanes 5-8) were electrophoretically separated by 8% SDS-PAGE, transferred to nitrocellulose and immunoblotted with EBA patient's sera (lanes 1-3 and 5-7) and normal human sera (NHS) (lanes 4 and 8).

The immunoreactivity of the newly expressed recombinant chimeric forms of collagen VII was analyzed by immunoblotting using sera from reference EBA patients and healthy donors. Representatives examples are shown in Figure 3. IgG autoantibodies from EBA patients' sera (n = 5) recognized the recombinant forms His-hCVII-NC1-NC2 (Figure 3, lanes 1-3) and His-hCVII-NC1-H-NC2 (Figure 3, lanes 5-7) of collagen VII. Normal human sera (n = 2) did not react with these recombinant forms of collagen VII (Figure 3, lanes 4 and 8).

### Development of ELISA using recombinant collagen VII

**Figure 4:**
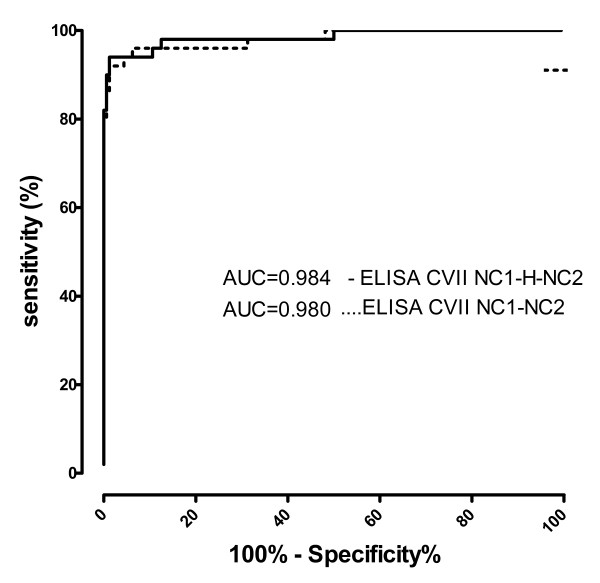
**Receiver-operating-characteristic (ROC) curve**. AUC, area under the curve. Test performed with sera from patients with epidermolysis bullosa acquisita (n = 50) and controls (n = 160).

The working conditions, including antigen amount/well, dilution of sera and secondary antibodies have been defined by an initial chessboard titration (data not shown). To determine the cut-off value of the newly established immunoassay, we performed a ROC analysis of the ELISA readings with sera from 50 EBA patients and 160 healthy donors for the ELISA results obtained with both His-hCVII-NC1-NC2 and His-hCVII-NC1-H-NC2. The area under the curve (AUC) was 0.980 (95% CI.: 95%-100%) and 0.984 (95% CI.: 96%-100%) for His-hCVII-NC1-NC2 and His-hCVII-NC1-H-NC2, respectively (Figure 4). Based on a calculated specificity of 97.50% and a sensitivity of 92% (His-hCVII-NC1-NC2) and 94% (His-hCVII-NC1-H-NC2) the cut-off was set at 0.425 and 0.322 OD reading units for His-hCVII-NC1-NC2 and His-hCVII-NC1-H-NC2, respectively.

### ELISA using recombinant NC1-NC2 and NC1-hinge-NC2 forms of collagen VII allows for a sensitive and specific detection of antigen-specific autoantibodies

**Figure 5:**
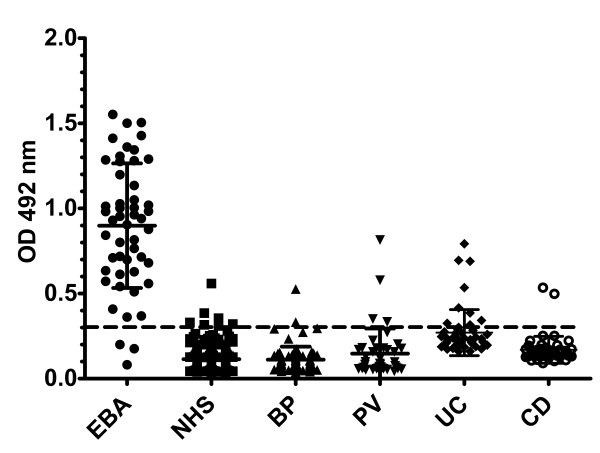
**ELISA reactivity of human sera with the recombinant NC1-hinge-NC2 noncollagenous domains of collagen VII**. Scatter plots represent optical density measurements of serum reactivity of epidermolysis bullosa acquisita (EBA), bullous pemphigoid (BP), pemphigus vulgaris (PV), Crohn's disease (CD), ulcerative colitis (UC) patients and healthy donors (NHS) with the purified recombinant chimeric collagen VII_. _(His-hCVII-NC1-H-NC2). The cut-off of the assay is represented by a dotted line.

Applying the cut-off value of 0.322 defined by ROC analysis for the newly developed ELISA showed that 47 EBA (94%; 95% CI: 87%-100%; n = 50), 2 CD (4%; 95% CI: 0%-9.43%; n = 50), 8 UC (16%; 95% CI: 5.8%-26%; n = 50), 2 BP (2.63%; 95% CI: 0%-6.23%; n = 76), 4 PV (9.52%; 95% CI: 0%-18.4%; n = 42) patients and 4 of the healthy donors (1.63%; 95% CI: 0%-3.21%; n = 245) showed IgG reactivity against the chimeric NC1-hinge-NC2-hCVII protein (Figure 5). Therefore, a sensitivity and a specificity of 94% (95% CI: 83.4%-98.75%) and 98%(95% CI: 94%-100%), respectively, were calculated for the ELISA detecting collagen VII-specific IgG autoantibodies in patients with EBA. The area under the curve (AUC) was 0.984 (95% CI: 96.3%-100%) indicating an excellent discriminatory power. The accuracy of the ELISA using only the NC1-NC2 domains of collagen VII was only slightly lower as demonstrated by a sensitivity of 92% (95% CI: 80.7%-97.7%) and a specificity of 97.50% (95% CI: 93.7%-99.3%) with an AUC of 0.980 (data not shown). The immunoassays using the 2 recombinant forms of collagen VII correlated well regarding their capacity to detect specific autoantibodies (r = 0.95; p < 0.0001).

### IgG levels by hCVII ELISA correlate with the IgG reactivity against the dermal-epidermal junction by IF microscopy

The indirect IF microscopy on salt-split skin is a standard diagnostic and monitoring tool in autoimmune bullous diseases. To further characterize the suitability of the newly developed ELISA for diagnosis of diseases associated with autoimmunity against collagen VII, we correlated the IgG levels by ELISA with the end-point titers by IF microscopy on salt-split skin in sera from patients with EBA (n = 9). When the IgG levels of collagen VII-specific IgG autoantibodies were plotted against the IgG titers measured by indirect IF microscopy, a positive correlation (r = 0.73; p < 0.05) was obtained. Interestingly, all sera from patients with BP (n = 2), PV (n = 4), CD (n = 2) and UC (n = 8) as well as from healthy donors (n = 4), which showed low levels of IgG autoantibodies against collagen VII by ELISA, did not show binding to the dermal side by indirect IF microscopy on salt-split skin.

### Levels of autoantibodies against collagen VII do not correlate with inflammation markers in inflammatory bowel disease

C-reactive protein (CRP) is routinely used as marker of disease activity in patients with IBD, especially in CD. To address a possible direct role of collagen VII-specific autoantibodies in pathogenesis of IBD, the ELISA levels of autoantibodies were correlated with the CRP values of the patients at the time of blood collection. The calculated correlation coefficients were r = 0.135 (p = 0.356) and r = -0.174 (p = 0.231) for UC and CD patients, respectively.

### IgG4 autoantibodies dominate the autoimmune response against collagen VII

**Figure 6:**
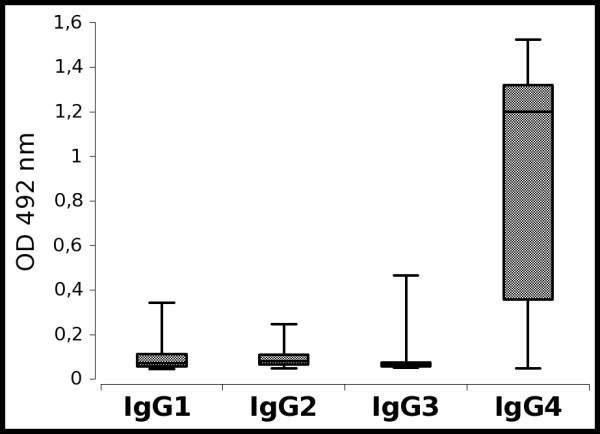
**Autoantibodies against collagen VII are mainly of IgG4 isotype**. Box-and-whiskers graphs represent descriptive summaries of the measurements for IgG1, IgG2, IgG3 and IgG4 autoantibodies against collagen VII by ELISA as described in Methods.

The IgG subclass of collagen VII-specific autoantibodies in patients' sera was analyzed by ELISA using recombinant His-hCVII-NC1-H-NC2 substrate. In 34%, 37%, 16% and 100% of sera autoantibodies of IgG1, IgG2, IgG3, and IgG4 isotype recognized the recombinant autoantigen (Figure 6). By correlating the results obtained for the IgG subclasses with the values obtained by the standard assay, correlation coefficients of r = -0.07 (p = 0.8), r = 0.17 (p > 0.1), r = 0.24 (p > 0.1) and r = 0.727 (p < 0.001) resulted for the detection of IgG1, IgG2, IgG3 and IgG4, respectively.

## Discussion

Autoimmune phenomena were observed in the development of both cellular and humoral responses. In fact, there may be significant overlap between the autoreactive and protective antibodies since polyreactive antibodies represent a substantial part of the normal repertoire. Antibodies against specific self-antigens are typically associated with systemic or organ-specific autoimmune diseases, but may be also found in neoplastic diseases and even in healthy subjects. EBA is a prototypical organ-specific autoimmune disease affecting the skin and the mucous membranes associated with autoantibodies against collagen VII [1]. The blister-inducing potential of collagen VII-specific autoantibodies has been shown in different *ex vivo *and animal models [13]. Autoimmunity against collagen VII has been described in IBD, which may be clinically associated with EBA [2]. Interestingly, collagen VII-specific autoantibodies are present in IBD patients, which do not show blistering skin disease [2]. The aim of the present study, was to analyze collagen VII-specific autoantibodies, including IgG subclasses in large groups of healthy blood donors or patients with autoimmune blistering diseases.

The rapid and accurate routine diagnosis of most autoimmune diseases relies on the detection of autoantibodies with known molecular specificity. Several immunoassays have been developed so far for the detection of autoantibodies against collagen VII (Table 2) [22,25-27]. To further improve the detection of collagen VII-specific autoantibodies, in the present study we have generated a chimeric antigen substrate containing virtually all autoepitopes, which have been reported in previous epitope mapping studies in patients.

**Table 2:** Sensitivity and specificity of ELISA systems for the detection of autoantibodies against collagen VII

Study	Recombinant autoantigen	Commercially available	Sensitivity	Specificity	Reference
Chen *et al. *(n = 24)	NC1 domain	No	100%	-	[22]

Pendaries *et al. *(n = 41)	Collagen VII full length	No	68%	96%	[25]

Saleh *et al. *(n = 49)	NC1 + NC2	Yes	93.8%	98.1%	[26]

Komorowski *et al.*(n = 73)	NC1 domain	Yes	91.8%	99.8%	[27]

Licarete *et al.* (n = 50)	NC1-hinge-NC2	-	94%	98%	present study

Wet lab epitope mapping studies and our present *in silico *analysis have shown that the epitopes targeted by autoantibodies are localized within the NC1, NC2 and most probably the hinge region of the antigen [19-22]. Therefore, for measuring autoantibodies against collagen VII we have generated a chimeric protein containing all putative epitope-bearing regions of collagen VII, including its NC1 and NC2 domains and the hinge fragment. The chimeric protein, which was produced in a human cell line to ensure optimal posttranslational modifications, shows an increased yield and is more stable compared with the full-length collagen while containing all the epitopes/regions in one copy per molecule. Thus, its use as a substrate does not require preadsorption against bacterial proteins and ensures strict equimolar concentration of the NC1, NC2 and hinge regions.

Interestingly, our *in silico *analysis predicted the existence of both linear and conformational epitopes. Since immunoblotting is not quantitative and uses denatured antigen, to measure autoantibodies against both linear and conformational epitopes on collagen VII, we have used ELISA.

Possible isolated reactivity against hinge was reported so far only in 3 children with EBA [21] and was apparently present in our relatively large cohort of adult patients only in one patient. This resulted in a slightly lower specificity of the ELISA using the fused NC1 and NC2 domains when compared with the form containing NC1, hinge and NC2 in the present study. The percentage of EBA patients with autoantibodies targeting only epitopes outside its NC1 and NC2 domains is unknown. However, since EBA was generally previously diagnosed by reactivity with the NC1 domain of collagen VII, patients showing only reactivity against the hinge region may have been largely excluded from our study. Therefore, the immunossay described here allows characterizing the prevalence of autoantibodies against the hinge region of collagen VII and should increase the yield of EBA patients, which will test positive by ELISA. In addition, as suggested by previous studies [21,35], the reactivity against the hinge region of collagen VII may be associated with inflammatory rather than mechanobullous blistering disease. The new recombinant proteins generated in this study will help clarifying this aspect in larger cohorts of patients with disease associated with autoimmunity against collagen VII.

Low levels of collagen VII-specific autoantibodies were detected in a number of patients with other autoimmune blistering diseases and healthy subjects. In patients with autoimmune and inflammatory diseases the presence of autoantibodies against collagen VII may be the result of an epitope spreading process. Our findings are in line with the observation that healthy blood donors and patients without skin blisters show pemphigoid autoantibodies [36-38]. The pathogenic significance of collagen VII-specific autoantibodies in other autoimmune blistering diseases and in healthy subjects is still unclear. As shown in our present study, these autoantibodies do not show binding to the dermal-epidermal junction by indirect IF microscopy suggesting that they do not bind *in vivo*. In addition, our previous *in vivo *studies documented that the mere presence of tissue-bound autoantibodies in experimental EBA does not result in skin disease [11].

Autoantibodies with different molecular specificity are present in healthy individuals and in patients with various diseases (e.g., ANAs prevalence is about 3-15%). We have found collagen VII-specific autoantibodies in 4% and 18% of patients with CD and UC, respectively. Our present results in CD patients are in line with our previous study showing collagen VII-specific autoantibodies by immunoblotting in 5.8% of CD and 5.8% of UC patients, in contrast to over 60% of CD patients initially reported [23,24]. Interestingly, we measured collagen VII-specific autoantibodies in a higher percentage of UC patients compared with 5.8% and 12.9%, that were reported in the previous studies [39,40]. The reason for this discrepancy is not known and future studies in larger number of IBD patients should help defining the prevalence of collagen VII-specific autoantibodies in these patients. There is apparently no correlation of collagen VII-specific autoantibodies with inflammation markers in patients with CD and UC. While several hypotheses have been advanced, the induction of autoimmune response against collagen VII and the pathogenic significance of specific autoantibodies in inflammatory bowel disease is still elusive [2,41].

As with other autoantibody-induced diseases, ELISA levels of collagen VII-specific autoantibodies likely correlate with the disease severity [13,26]. It is therefore expected that measuring the autoantibody levels, will help predicting short-term clinical evolution in EBA patients and guide therapeutic decisions. A more general prognostic value of the levels of EBA autoantibodies for the long-term disease course, including the resistance to treatment, has not yet been addressed. Addressing this question, which requires a more wider approach in prospective clinical study, should be addressed in the future using the tools generated this study.

Our present results strongly suggest the existence of non-pathogenic autoimmunity against collagen VII in patients and healthy individuals. Why autoantibodies specific to collagen VII and XVII do not induce tissue damage in all individuals and conditions is not known. Inadvertent autoimmune responses may be uncoupled from disease by various mechanisms, including cryptic B cell autoepitopes, typically seen in Goodpasture syndrome [39,40], anatomic, cellular and molecular barriers that avert either tissue deposition of immune complexes [42,43] or the engagement of inflammatory effectors by tissue-bound antibodies [44]. In this context, the IgG subclass is a major determinant of autoantibody pathogenicity. Similar to a previously published report, our IgG subclass analysis revealed a dominant IgG4 response in patients with collagen VII-specific autoantibodies [45]. However, while the other subclasses of autoantibodies were less represented we could not document a strict restriction to IgG1 and IgG4 [45]. The mechanisms of tissue damage in EBA may be inflammatory, requiring the activation of complement and leukocytes by bound autoantibodies, or non-inflammatory just involving the binding of autoantibodies independent of their Fc portions [13]. Our own results and data from the literature show that in addition to IgG1, which shows good Fcγ-dependent complement- and leukocyte-activating capacity, non-complement-fixing IgG4 autoantibodies contribute to tissue damage by activating the leukocytes, albeit with a reduced efficiency compared with IgG1 [46]. In addition, IgG4 could induce tissue damage just by binding to collagen VII in an Fc-independent manner [13]. While experimental data to support this hypothesis are still scarce, our present findings suggest that IgG4 may unfold its pathogenic potential in this way.

## Conclusions

In conclusion, we have developed an immunoassay using a chimeric recombinant collagen VII containing major *in silico *predicted and wetlab mapped autoepitopes for detecting autoantibodies against collagen VII. We show a low prevalence of collagen VII-specific autoantibodies in patients with unrelated inflammatory and autoimmune diseases. This immunoassay will be a useful tool for the sensitive and specific detection of collagen autoantibodies in epidermolysis bullosa acquisita and other diseases associated with autoimmunity against collagen VII.

## Competing interests

The authors declare that they have no competing interests.

## Authors' contributions

EL and CS designed and performed the ELISA, coordinated the data acquisition, analyzed and interpreted the data and drafted the manuscript. SG produced the recombinant protein, characterized its immunoreactivity and performed IgG subclass analysis by ELISA. MJR and CS performed the *in silico *analysis. GDZ, TH, MH, GZ, GH, JM, MFN and LBT provided serum samples used in the study and have participated in the experimental design and drafting of manuscript. All authors read and approved the final manuscript.

## Supplementary Material

Additional file 1 **Table S1 Predicted antigenic epitopes of human collagen VII**. Click here for file
