# Supplementary material for: Prevalence of collagen VII-specific autoantibodies in patients with autoimmune and inflammatory diseases
Source: BMC Immunol. 2012 Apr 4;13:16. doi: 10.1186/1471-2172-13-16 (PMC3368718; doi:10.1186/1471-2172-13-16)
Supplement: Additional file 1 — Table S1 Predicted antigenic epitopes of human collagen VII. [file 1471-2172-13-16-S1.PDF]

Supplemental material

Supplemental Table 1. Predicted antigenic epitopes of human collagen VII.

Start - End	Score	Sequence	Region
4 - 24	1.239	RLLVAALCAGILAEAPRVRAQ	NC1
121 - 148	1.225	AAILHVADHVFLPQLARPGVPKVCILIT	NC1
1244 - 1254	1.223	PEPCPVYCPKG	NC1
2826 - 2839	1.214	SQLHAVPVLRVSHA	NC2
660 - 669	1.213	TYQVAVSVLR	NC1
748 - 802	1.193	EYTVHVRAHVAGVDGPPASVVVRTAPEPVGRVS RLQILNASSDVLRITWVGVTGA	NC1
1154 - 1168	1.193	RQHVPGVMVLLVDEP	NC1
455 - 469	1.192	EPPQKVVLPSDVTRY	NC1
1038 - 1061	1.191	PEASVTQTPVCPRGLADVVFLPHA	NC1
291 - 313	1.186	SVRLRGLRPLTEYQVTVIALYAN	NC1
855 - 863	1.184	PVSIVVTTP	NC1
2885 - 2911	1.183	CTAYTLRWYHRAVTGSTEACHPFVYGG	NC2
836 - 849	1.178	GGVSYSVRVTALVG	NC1
1072 - 1109	1.177	TRRVLERLVLALGPLGPQAVQVGLLSYSHRPSPL FPLN	NC1
1541 - 1552	1.174	DPAVVGPAVAGP	C
597 - 611	1.173	TPLAVPGLRVVVSDA	NC1
2926 - 2937	1.171	ERRCPPRVVQSQ	NC2
192 - 227	1.169	SDFFFFVNDFSILRTLLPLVSRRVCTTAGGVPVTR P	NC1
675 - 683	1.168	PAAVIVART	NC1
865 - 879	1.165	EAPPALGTLHVVQRG	NC1
340 - 353	1.161	AHSLLVAWRSVPGA	NC1
686 - 707	1.157	LGPVRTVHVTQASSSSVTITWT	NC1
481 - 514	1.152	RLTLYTLLEGHEVATPATVVPTGPELPVSPVTDL	NC1
1012 - 1035	1.152	SQRVTGLEPGVSYIFSLTPVLDGV	NC1
26 - 46	1.151	RERVTCTRLYAADIVFLLDGS	NC1
2799 - 2822	1.148	SQHCACQGQFIASGSRPLPSYAAD	NC2
2299 - 2306	1.148	GQAVVGLP	C
2750 - 2761	1.146	ERVVGAPGVPGA	C
929 - 939	1.146	QYRVRLSVLGP	NC1
585 - 592	1.145	SASVLTVR	NC1
165 - 176	1.139	GQGVKLFAVGIK	NC1

562 - 579	1.139	LDDVQAGLSYTVRVSARV	NC1
1222 - 1239	1.138	LDQAVSGLATALCQASFT	NC1
1577 - 1584	1.136	PGLVLPGD	C
57 - 72	1.136	VRSFLEGLVLPFSGAA	NC1
392 - 406	1.134	EVTVSTLFGRSVGPA	NC1
1210 - 1219	1.134	VQTFFAVDDG	NC1
2874 - 2883	1.131	DPCSLPLDEG	NC2
955 - 990	1.129	PRVPSIELRVVDTSIDSVTLAWTPVSRASSYILSW R	NC1
548 - 557	1.128	ERTLVLPGSQ	NC1
377 - 385	1.126	QGSVLLRDL	NC1
416 - 441	1.126	SVEQTLRPVILGPTSILLSWNLVPEA	NC1
1135 - 1149	1.125	GTAVVTAHRYMLAPD	NC1
2522 - 2529	1.122	SAVILGPP	C
235 - 243	1.117	PRDLVLSEP	NC1
521 - 534	1.116	GQRVRVSWSPVPGA	NC1
2631 - 2637	1.112	KGACGLD	NC1
1182 1191	1.106	ASGLNVVMLG	NC1
1968 - 1974	1.105	GSFLPVP	hinge
2384 - 2406	1.105	PPGVKGDLGLPGLPGAPGVVGFP	C
613 - 624	1.104	RVRVAWGPVPGA	NC1
74 - 85	1.103	AQGVRFATVQYS	NC1
256 - 278	1.102	SGPVTGYKVQYTPLTGLGQPLPS	NC1
2091 - 2098	1.101	GPKVSVDE	C
359 - 365	1.100	TWRVLSG	NC1
908 - 924	1.098	QSRVLGPELSSYHLDGL	NC1
882 - 901	1.095	SLRLRWEPVPRAQGFLLHWQ	NC1
2372 - 2379	1.093	GVGVPGSP	C
1113 - 1120	1.093	DLGIILQR	NC1
536 - 544	1.092	QYRIIVRST	NC1
1937 - 1949	1.085	GSVPNVDRLLETA	C, hinge
1951 - 1959	1.084	IKASALREI	hinge
714 - 723	1.082	GYRVSWHSAH	NC1
2223 - 2232	1.082	TGAVGLPGPP	C
2274 - 2289	1.082	PGVPGSPGLPGPVGPK	C
1888 - 1898	1.082	PPGLPGPVGPP	C

2319 - 2326	1.080	AGDLVGEP	C
245 - 253	1.080	SQSLRVQWT	NC1
153 - 163	1.079	QDLVDTAAQRL	NC1
737 - 743	1.077	VAELDGL	NC1
183 - 189	1.075	LKRVASQ	NC1
2243 - 2249	1.075	SPGLPGQ	C
2234 - 2241	1.074	PSGLVGPQ	C
1662 - 1668	1.074	APGVRGP	C
728 - 735	1.072	SQLVSGEA	NC1
1425 - 1431	1.063	LPGLPGS	C
2016 - 2022	1.063	PPGLALG	C
2574 - 2586	1.062	AGLPGLRGLLGPQ	C
2855 - 2863	1.062	YSEYSVEEY	NC2
1352 - 1358	1.061	PGQVIGG	C
1624 - 1629	1.055	PGPVGP	C
1508 - 1514	1.055	LPGVAGR	C
1705 - 1712	1.055	PGRLVDTG	C
997 - 1010	1.054	QEVPGSPQTLPGIS	NC1
100 - 110	1.052	GGDVIRAIREL	NC1
1880 - 1886	1.049	PGILGPQ	C
2436 - 2442	1.049	PGPLGPP	C
1435 - 1441	1.048	QGPVGPP	C
2444 - 2451	1.048	PPGSVGPP	C
1265 - 1271	1.048	RGQVGPP	C
640 - 646	1.047	SQTLPPD	NC1
2190 - 2197	1.036	APGLAGPA	C
2176 - 2182	1.034	PGPVGGH	C
1174 - 1180	1.030	FSPIREA	NC1
1455 - 1460	1.029	PGLPGQ	C

[1]Antigenic sites with a score higher than 1 are shown; [2]NC1, noncollagenous domain 1; [3]NC2, noncollagenous domain 2; [4]C, collagenous domain
